# It’s more than dryness and fatigue: The patient perspective on health-related quality of life in Primary Sjögren’s Syndrome - A qualitative study

**DOI:** 10.1371/journal.pone.0172056

**Published:** 2017-02-09

**Authors:** Angelika Lackner, Anja Ficjan, Martin H. Stradner, Josef Hermann, Julia Unger, Tanja Stamm, Georg Stummvoll, Mona Dür, Winfried B. Graninger, Christian Dejaco

**Affiliations:** 1 Division of Rheumatology and Immunology, Department of Internal Medicine, Medical University Graz, Graz, Austria; 2 University of Applied Sciences JOANNEUM, Department of Health Studies, Institute of Occupational Therapy, Bad Gleichenberg, Austria; 3 Division of Rheumatology, Medical University Vienna, Vienna, Austria; 4 IMC University of Applied Sciences Krems, Krems an der Donau, Austria; 5 Medical University of Vienna, Department of Pediatrics and Adolescent Medicine, Division of Neonatology, Pediatric Intensive Care and Neuropediatrics, Vienna, Austria; 6 Rheumatology Service, South Tyrolean Health Trust, Hospital of Bruneck, Bruneck, Italy; Brown University, UNITED STATES

## Abstract

**Objectives:**

In Primary Sjögren’s Syndrome (PSS), there is an apparent lack of data concerning the perspectives of patients, their needs, preferences and difficulties of daily life. This qualitative study was conducted to explore perspectives and needs of patients with PSS that influence health related quality of life (HRQL).

**Methods:**

We recruited 20 PSS patients fulfilling the American-European consensus classification criteria out of the PSS cohort of the Medical University Graz, Austria. In total, 6 focus group sessions (with three to four patients per group) were performed. A modified meaning condensation procedure was used to analyse the data.

**Results:**

The interview analysis resulted in 484 meaning units, 254 subconcepts and 86 concepts. The identified concepts were grouped into three dimensions: physical dimension, psychological & emotional challenges and social life & daily living. A dependency between the three categories was identified.

The concepts most commonly reported by patients were related to the physical dimension: pain and dryness as well as complaints associated with/provoked by these symptoms. Patients also reported shortness of breath, fatigue und constipation.

**Conclusions:**

This qualitative study underpins that HRQL in PSS patients is affected by several factors. The problems are not limited to dryness, pain and fatigue while the complaints secondary to these symptoms are important to patients with PSS significantly affecting physical, psychological and social life components of HRQL. A disease-specific patient related outcome measures for clinical practice and trials should be developed considering the different aspects of HRQL in PSS.

## Introduction

Primary Sjögren’s Syndrome (PSS) is one of the most common systemic autoimmune disorders affecting 0.3–5% of the general population [1–4]. PSS predominantly occurs in women (female: male ratio 9:1) and its incidence peaks in the fifth and sixth decade of life [5,6].

Inflammation of lacrimal and salivary glands results in decreased production of tears and saliva causing dryness of eyes, mouth, gastrointestinal and genital tract as well as skin. A high proportion of patients also experience extraglandular manifestations including fatigue, musculoskeletal, gastrointestinal and/or neurological symptoms [7]. Furthermore, PSS patients are at a higher risk of developing malignancies, particularly non-Hodgkin’s Lymphomaod [8–12]. Therapy of PSS is often symptomatic focusing on the management of sicca symptoms, pain and fatigue [13,14].

Previous studies reported that health related quality of life (HRQL) is impaired in PSS patients compared to the general population [15,16]. To our knowledge, this is the first qualitative study exploring the individual factors affecting HRQL in PSS.

Impaired HRQL may be understood as the overall burden caused by the disease and it’s treatment to patients’ daily individual and social life [17]. The most important dimensions of HRQL include overall physical and social function, burden of symptoms and emotional status and general life satisfaction [18]. A qualitative approach provides the opportunity to assess HRQL in a holistic way including patients’ perspectives and needs [17,19].

The purpose of this study was to explore the perspectives and aspects of HRQL in patients with PSS in a qualitative manner.

## Patients and methods

We conducted a qualitative study, guided by a phenomenological approach with focus group interviews to investigate the aspects of HRQoL important to PSS patients. Focus groups were preferred over individual interviews because we expected that the exploration of views and opinions through group discussion would provide more information and concepts than individual questioning [20].

### Participants

PSS patients treated at the rheumatology outpatient clinic of the Medical University of Graz were invited by phone to participate in this study. For patient sampling/selection, we followed the “maximum-variation strategy” considering disease duration and age in order to ensure that patients either with new-onset disease as well as with long-standing disease experience were included[21]. Data saturation was determined as the point when sufficient information about the study aim was obtained and no further concepts could be identified. All patients fulfilled the American-European consensus classification criteria [7]. This study was approved by the institutional review board of the Medical University Graz and written informed consent was obtained from all participants.

### Focus group interviews

All focus groups were chaired by the same moderator (AL) aided by one assistant (AF) responsible for observing the group, taking field notes and recording the data. The moderator and assistant did not have any pre-existing relationship with the participants, and they were employed as study coordinator and resident physician, respectively at the department of Rheumatology of the Medical University Graz. The moderator was well trained in qualitative research techniques, and she introduced herself at the beginning of the interviews. Interviews were in German language, and they were conducted in a quiet room at the rheumatology outpatient clinic of the Medical University of Graz.

For each focus group, a discussion guide was developed with an opening question (patients’ introduction with disease duration, beginning of the disease etc.) and four main open-ended questions based on the three (i.e. the physical, mental and social) dimensions of HRQL (21) and a further literature review (see Table 1 for topic guide).

**Table 1:** Topic guide used to maintain focus group discussions.

Topic guide
1. Could you introduce yourself and describe the way of your disease?
2. Which PSS-related problems do you experience and which parts of the body are involved? (e.g. Dryness of eyes, mouth, nose, vagina, skin? Side-effects of medication? Limitation through treatment?)
3. Do you experience any difficulties in your activities of daily living? (e.g. which kind of work? Sick leave? Household, hobbies and leisure activities?)
4. Do you experience any limitations in your social environment? (e.g. impairment of social contacts? Support from family/friends?)

At the beginning of each focus group session, the moderator explained the interview process, the aim of the study and started the discussion with interview question 1. Similar to other qualitative studies in rheumatology, the presumed duration of the focus-group discussion was about one hour [22,23].

All interviews were digitally recorded and transcribed verbatim; thereby the patients’ data were anonymised.

### Data analysis

**Fig 1:**
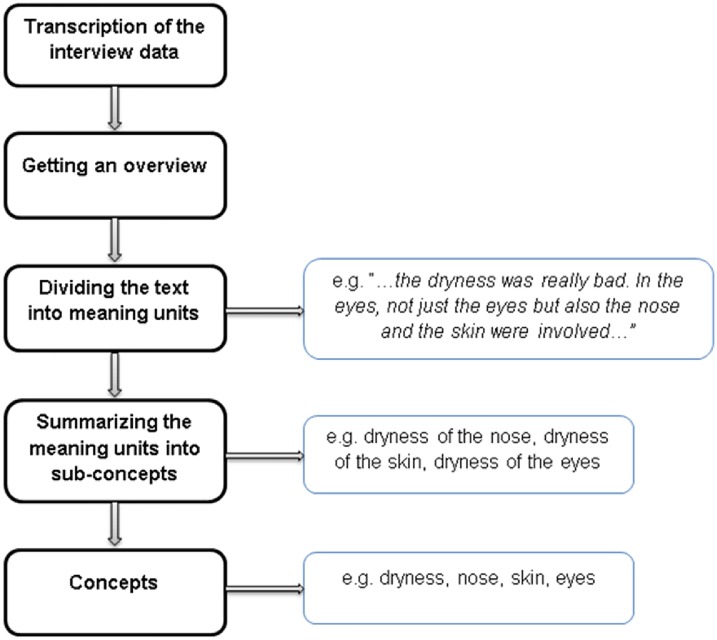
Process of data analysis.

A modified meaning condensation procedure was used to analyse the focus group sessions (Fig 1) [24]. All analyses were performed by the moderator (AL). The meaning condensation procedure enables a stepwise reduction of the transcribed text into short formulations reflecting interviewees’ meanings: First, the transcribed focus group interviews were read through to gain an insight into the data material. Second, the data material was divided into ‘meaning units’, which referred to a specific unit of text with a common phrase or a few words or a few sentences with a common meaning. Third, sub-concepts were identified among the meaning units which best reflected the meaning units. A meaning unit could contain more than one sub-concept. Fourth, the identified sub-concepts were grouped into more comprehensive concepts [24]. For the management of interview data and handling of the concepts, we used the software Atlas.Ti (Berlin, Germany) [25]. After analysis three interviewed patients checked and verified the resulted concepts. According to the aim of the study, to explore the aspects of HRQL, the identified concepts were assigned to the most appropriate main dimension of HRQL [18].

## Results

### Participants

Sixty-two patients were invited to this study and 20 (32.3%) finally agreed to participate. The reasons for declining study participation were lack of interest and/or time. Six focus group sessions with three (4 focus groups) or four (2 focus groups) participants each were conducted. Small focus groups of four to six participants were planned [24] but due to non-attendance focus groups were smaller than planned (three to four participants in each). Patients’ characteristics are summarized in Table 2. The average duration of each focus group session was 58 (SD ± 13) minutes.

**Table 2:** Patient characteristics.

Characteristic	Value
*Age, mean ± SD (years)*	62±8
*Disease duration, mean ± SD (years)*	5±2
*Symptom duration before diagnosis, mean ± SD (years)*	2.7±2.8
*Extraglandular symptoms, no. (%)*	
*Arthralgia/Arthritis*	6 (30)
*Raynaud phenomena*	5 (25)
*Lymphadenopathy*	4 (20)
*Depression*	4 (20)
*Fatigue*	16 (80)
*Monoclonal gammopathy of undetermined significance (MGUS)*	3 (15)
*Treatment no. (%)*	
*None*	11 (55)
*Pilocarpine*	2 (10)
*Pilocarpine*+*Chloroquine*	3 (15)
*Pilocarpine*+*Hydroxychloroquine*	1 (5)
*Chloroquin*	1 (5)
*Hydroxychloroquine*	1 (5)
*Corticosteroids*	1 (5)

### Concepts and sub-concepts identified in the qualitative analysis

**Fig 2:**
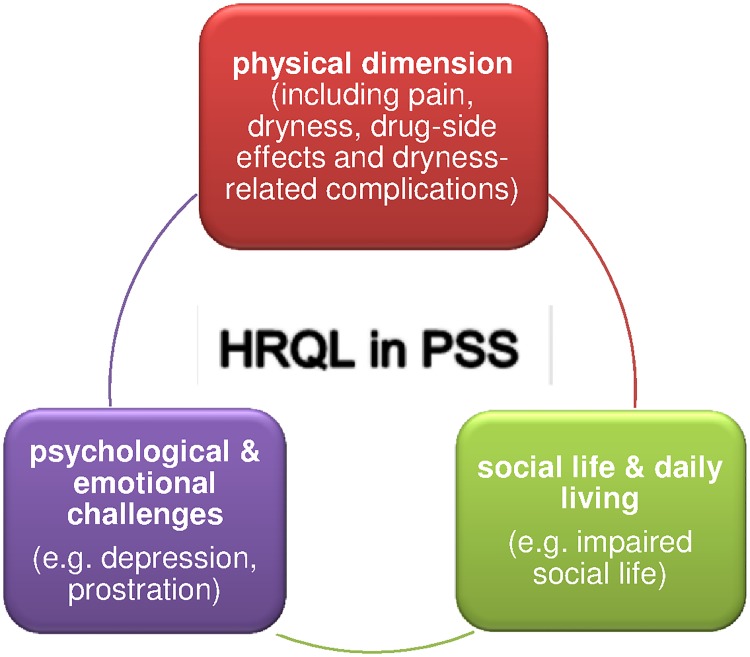
Three main themes of HRQL in PSS patients with their influence among each other.

Overall, we retrieved 484 meaning units. These were reduced to 254 sub-concepts and subsequently grouped into 86 concepts (Table 3). The concepts were then assigned to three dimensions: physical, psychological & emotional as well as social life & daily living (Fig 2) [18]. These three dimensions reflect the different aspects of health related quality of life in PSS according to the patients’ perspective.

**Table 3:** The identified concepts of HRQL in PSS patients.

Concepts		
Physical dimension	Psychological & emotional challenges	Social life and daily living
**Pain**	fear of physician fear of side effects feeling of being an encumbrance for relatives Loneliness Hobby Prostration worries about the future living with a chronic disease long way until diagnosis standard of living decreased performance Complaints were dismissed by health professionals psychological stress (chronic dryness and getting dismissed) Worsening of complaints when stressed suicidal ideation excessive demands Getting dismissed impaired self-confidence dissatisfaction with treatment lack of understanding for complaints impossibility to cry additional stress with family	Impaired social life Family is considerate of patient dependency on relatives in daily life Difficulties at work(computer, speaking, fatigue) working despite feeling sick fear of unemployment different specialists difficulties at driving a car Sports household activities limitation in nutrition Eating disturbances financial dependency financial stress hobby walking disease education Dissatisfaction with own appearance aids for dryness complaints depend on season inability to apply make-up Hospitalization drug efficacy physical therapy complementary medicine
Joints (swelling & stiffness) back/spine extremities stomach wandering pain pain due to dryness		
**Dryness**		
eyes mouth skin nose ears vagina		
**Additional physical complaints**		
sensitive to coldness lymphoma arthrosis shortness of breath loss of muscle power swelling of lymph nodes fatigue Raynaud syndrome photophobia Constipation Depression		
**Dryness-induced complaints**		
burning in the mouth inflammation of eyes &ears reduced sense of smell reduced sense of taste loss of weight Inability to cry gritty eye sensation worsening of vision speaking Loss of teeth sleeping disturbances due to dryness of mouth and eyes inability to eat and chew drinking compulsion at night		
**Side effects**		
hair loss excessive sweating sight disorder		

Most commonly reported concepts were related to the physical dimension (although some concepts could have been assigned to more than one dimension): pain, dryness and complaints secondary to dryness such as painfully dry mucosae, recurrent inflammation of the eyes and ears, sleeping disturbances and the inability to eat and chew. Patients also frequently reported joint pain, shortness of breath, fatigue und constipation.

Complaints related to dryness were not limited to the physical dimension. Patients reported for example, that dryness of the oral mucosa resulted in a burning sensation in the mouth, loss of senses of smell and taste, loss of weight, inability to eat and chew food as well as speaking difficulties. These complaints subsequently led to psychological & emotional challenges because of a perceived lack of understanding by relatives as well as difficulties at work, limitation of nutrition and impaired social life. Furthermore, patients’ perceived dryness of eyes caused photophobia, inflammation of the eyes, inability to cry, gritty eye sensation, impaired sight, impossibility to drive a car or work on the computer and financial burden due to expensive eye drops.

The three categories and examples for their influence are discussed below, including supporting quotes (G = Group, I = Interviewee).

#### Physical dimension

The physical dimension revealed the most concepts (n = 38). Among them, five major subcategories were identified: pain, dryness, drug-side effects, dryness-related complications and additional complaints.

Patients reported pain of joints, wandering muscle pain and pain due to dryness, as expressed by the following statements:

G4, I4: ‘Every week I have pain somewhere else. I mean, the whole spine, feet, the knee. Sometimes I think I’m going crazy. Every week it hurts somewhere else. And I don’t know why.’

G4, I3: ‘Yes, when it doesn’t hurt in the morning, I’m thinking that I’m not alive.’

G4, I2: ‘Every week it hurts somewhere else and you think that can’t be real. The limbs, everything hurts.’

As stated above, dryness caused several secondary complaints. One female patient expressed problems with eating as follows (G4, I4):

‘The whole time I have a bottle of water with me. But, I can’t eat some dry food. You can’t swallow it. You have the feeling that you are suffocating.’

The dryness of mouth also caused sleeping disturbances (G1, I3):

‘Sleeping was just possible, when I was sitting in bed. As soon as I turned around, I had a feeling of suffocation. So I was sleeping in a sitting position for one and a half year.’

The most commonly reported drug side-effect was excessive sweating after intake of pilocarpine, which was the main reason why patients stopped taking this drug.

As an example of additional physical complaints, patients experienced sensitiveness to coldness, loss of muscle power, constipation, fatigue and prostration.

#### Psychological & emotional challenges

Many patients reported about their experiences in the initial phase of the disease, when the diagnosis was not yet established. For most patients, it took several years before they were finally referred to a rheumatologist. They reported that (mainly family) physicians frequently suspected them as having a mental/psychiatric disorder or simply ignored their complaints.

One patient described her experience (G3, I1):

‘I had problems with my stomach and digestion, felt pain in my joints. The physician did not find a reason for that. After a while, I also had dry eyes with burning and pain. I went to my general practitioner but he did not take me seriously. Then I recognised that my mouth became dry. I was afraid to go to a doctor because they looked at me, as being crazy myself. They wanted to send me to a psychologist. Then, after 5 years of symptoms and unsatisfying visits at different doctors, one doctor was on the right way and I finally got the diagnosis of Sjögren’s’s. I really felt relieved.’

One patient reported how the inability to cry emotionally challenged her (G2, I4):

‘I’m starting to cry tears, which I don’t have. In former times, when I had problems, I was able to cry and the problems were easier to handle. Now, I want to cry, because I have this disease and pain but I don’t have tears, and the feeling of crying is going by. I just want to cry.’

The feeling of being an encumbrance for family, excessive demand due to complaints and worries about the future were important aspects in this dimension. Most patients recognised that their complaints worsened when they suffered additional stress with family and/or job.

#### Social life & daily living

PSS patients felt a dependency on relatives in daily life, e.g. most patients were not able to drive a car because of dry eyes or they could not handle the household without help because of pain. Dryness caused difficulties at work particularly in case of a computer work and patients felt that long conversations with customers were challenging. They worked despite feeling sick and were afraid of losing their job.

Fatigue was a common reason for impairment of social life as expressed by the following statements:

(G2, I2) ‘Yes I can say that I’m often really tired, when friends are calling me; they want to go out after work. But I don’t have enough energy. I don’t want. Then I’m done. Honestly, I’m just happy when I can lay down. I do not have any energy anymore.’

(G5, I2) ‘My husband has adjusted to this situation. But when you are never able to do anything together and you are not fit enough (…) You have to take care. You have to take care because otherwise you will be alone. And the others are living their lives. It is like that. And in the family (…) that hurts.’

(G5, I2) ‘ (…) But now everything is so painful. I can’t go out in the evening. I miss that. I mean, my life is really impaired. Really impaired. And the others can’t understand that. They go riding a bike and you can’t go with them. Yes, then you are alone.’

The most frequently mentioned concepts related to therapy within this dimension included the relevance, efficacy and costs of complementary medicine, conventional drugs, physical therapy and aids for symptoms of dryness. It was particularly emphasized by patients that these therapies caused a significant financial burden.

## Discussion

To our knowledge, this project with focus-group interviews is the first qualitative study to explore the experiences and perspectives concerning HRQL in patients with PSS.

Pain, dryness and the consequences of dryness (inflammation of eyes and ears, loss of sense of smell and taste etc.) were the concepts most commonly raised by patients [26]. Although these factors belong mainly to the physical dimension, they were also relevant to the psychological/emotional as well as for patients’ social life: Several patients for example were “worried about the future” of their disease, “felt of being an encumbrance for their families” (dimension of social life & daily living). In addition, the symptoms of the disease caused “dependency on relatives in daily life”, “difficulties at work” and “financial burden” (social life & daily living). In daily clinical practice, physicians may currently not pay enough attention to (or even ignore) these aspects of disease because of time constraints and/or lack of awareness. To patients, however, these factors appear to be equally important than physical complaints and should thus be assessed in a systematic manner.

Most of the identified concepts belonged to the physical dimension—that was also found in other rheumatic conditions (Psoriatic Arthritis, Systemic Sclerosis, Rheumatoid Arthritis (RA), Systemic Lupus Erythematodes (SLE) and Hand Osteoarthritis) [27]. HRQL of PSS is comparable to that of patients with SLE, RA and Fibromyalgia, as measured by the generic Short Form-36 questionnaire [28,29].

The concept of “inability to cry” was previously reported by another study suggesting that PSS patients, due to the loss of tears, may use other ways of expressing emotions [30].

Another important emotional burden for PSS patients was the long lag from symptom to diagnosis as well as the experience of not being taken seriously by family practitioners and other physicians in the pre-diagnostic phase [31]. Even if a causal (or even effective symptomatic) therapy is not available yet, many patients felt relieved after having been diagnosed with PSS, given that finally they had an explanation for their complaints. Educational programs increasing the awareness of PSS among general practitioners, ophthalmologists, dentists and other specialists might support an early recognition of the disease and thus reduce the emotional stress of patients in the early phase of the disease. The concepts of “a long way until diagnosis” and not “being taken seriously” were also retrieved in qualitative studies of SLE [26] and Systemic Sclerosis [32]. The low level of experience of family physicians with these diseases as well as the wide range of unspecific symptoms might explain this observation. Moreover, there is often a discordance between subjective impairments and objective tests presumably causing misunderstandings and disagreements between physicians and patients concerning the relevance of dryness, fatigue, malaise and other symptoms [33]. Occupational therapy could have a positive impact on coping with PSS symptoms like sleep, pain, fatigue and impairment of daily activities as suggested previously [34]. Another interesting observation is variable perception of the necessity of treatment and medical visits by PSS patients. The majority of patients (at least in the early phase of the disease) desire treatment for symptom relief [35]. The efficacy of current therapies, however, is low to moderate at best [14], and therefore, most patients have developed their own coping strategies to better manage the disease. Some patients questioned the necessity of clinical visits, because “nothing is getting better, so why should we go to the doctor?” On the other hand, many patients were also concerned about the disease course (and complications) and therefore still preferred a regular medical control [36].

The reliable assessment of patients’ outcomes and particularly HRQL in clinical practice as well as treatment studies is an unmet need in PSS. A few patient-derived outcome measures have been developed for the assessment of dryness, fatigue and pain in PSS [8,37]; however, there is no tool for the assessment of PSS related HRQL available yet. Disease specific measures of HRQL have been developed for other rheumatic diseases already such as Rheumatoid Arthritis [38] or Psoriatic Arthritis [39] and the present study might be used as the basis for such an exercise in PSS.

An important limitation of our study is that the sample included only patients from one region in Austria, although patients were from different age groups and professional backgrounds. We only included female patients because none of our (few) male patients wanted to participate in this study [5]. A limitation of focus groups is the potential shyness of participants to talk about sensitive topics in front of other, mostly unknown people. Therefore, we cannot exclude that full data enrichment has not been obtained.

In summary, pain, dryness and complaints secondary to these symptoms are important to patients with PSS significantly affecting physical, psychological and social life components of HRQL. A tool for the systematic assessments of HRQL in PSS patients is needed for clinical practice and trials, and the results of this study might be used for the development of such a tool.
